# Genome-wide acceleration of protein evolution in flies (Diptera)

**DOI:** 10.1186/1471-2148-6-7

**Published:** 2006-01-25

**Authors:** Joël Savard, Diethard Tautz, Martin J Lercher

**Affiliations:** 1 Institut für Genetik der Universität zu Köln, 50674 Köln, Germany; 2 Department of Biology and Biochemistry, University of Bath, Bath BA2 7AY, UK; 3 European Molecular Biology Laboratory, 69012 Heidelberg, Germany

## Abstract

**Background:**

The rate of molecular evolution varies widely between proteins, both within and among lineages. To what extent is this variation influenced by genome-wide, lineage-specific effects? To answer this question, we assess the rate variation between insect lineages for a large number of orthologous genes.

**Results:**

When compared to the beetle *Tribolium castaneum*, we find that the stem lineage of flies and mosquitoes (Diptera) has experienced on average a 3-fold increase in the rate of evolution. Pairwise gene comparisons between *Drosophila *and *Tribolium *show a high correlation between evolutionary rates of orthologous proteins.

**Conclusion:**

Gene specific divergence rates remain roughly constant over long evolutionary times, modulated by genome-wide, lineage-specific effects. Among the insects analysed so far, it appears that the *Tribolium *genes show the lowest rates of divergence. This has the practical consequence that homology searches for human genes yield significantly better matches in *Tribolium *than in *Drosophila*. We therefore suggest that *Tribolium *is better suited for comparisons between phyla than the widely employed dipterans.

## Background

Understanding the causes of rate variation in protein evolution is central for many fields including molecular evolution, comparative genomics and structural biology. A widely accepted principle is that more important proteins evolve more slowly [1]. However, evolutionary rate variation not only exists between different proteins, but also between lineages [2]. This was already observed in the earliest comparative studies, both within [3-5] and across animal phyla [6].

Following the initial observation that Drosophilids are fast evolving [6], it was shown that the rate of synonymous substitutions in *Drosophila melanogaster *is approximately two times higher than in rodents and ten times higher than in primates [7]. However, the estimated acceleration seemed to depend on the types of proteins examined, with another study reporting only a 3-fold difference between *Drosophila *and mammalian rates [8]. A study of the relative rates of ribosomal RNA evolution in insect lineages showed that there was an episodic substitution rate increase of about 20-fold in the stem lineage of dipterans [9], suggesting that high evolutionary rates may be characteristic of the whole dipteran order. However, it is currently unclear whether the observed rate accelerations encompassed the whole nuclear genome, or whether they were restricted to certain classes of genes.

In this study, we assess the genome-wide variation of evolutionary rates between insect lineages, by comparing the beetle *Tribolium castaneum*, and the dipterans *Drosophila melanogaster *and *Anopheles gambiae*. We test (i) whether there is an acceleration of protein evolution in dipterans that is observable on a genome-wide scale, (ii) whether this acceleration is confined to an episodic burst of changes at the base of the dipteran lineage, and (iii) whether this acceleration affects all genes to a similar extent.

## Results

### Genomic rate estimates confirm an acceleration in dipterans

**Figure 1:**
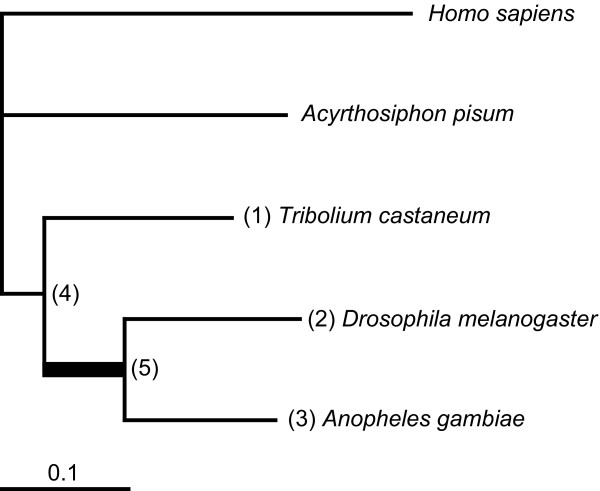
**Phylogenetic tree topology and maximum likelihood branch length estimates**. Numbers in parentheses indicate nodes used to measure distances in Table 1. The thick branch between nodes 4 and 5 represents the dipteran stem lineage.

To estimate genomic evolutionary rates of insect lineages, we formed 439 protein clusters derived from cDNA or EST data from beetle (*Tribolium castaneum*), fly (*Drosophila melanogaster*), mosquito (*Anopheles gambiae*), aphid (*Acyrthosiphon pisum*), and human (*Homo sapiens*). Concatenation of these orthologous sequences resulted in a single, well-defined alignment of 64,134 amino acids. Given the known tree topology in Figure 1[10], branch lengths were estimated using a maximum likelihood method [11] (Table 1). The dipteran branches are much longer than the *Tribolium *branch, indicating accelerated evolution in the Diptera. Since their last common ancestor, *Drosophila *has accumulated 36% (95% CI: 31–42%) more amino acid substitutions compared to *Tribolium*, while the corresponding increase for *Anopheles *compared to *Tribolium *is 23% (95% CI: 18–29%).

**Table 1:** Relative evolutionary rates of *Tribolium castaneum*, and the dipterans *Anopheles gambiae *and *Drosophila melanogaster*. Amino acid distances, divergence time estimates, and substitution rates for the holometabolous insect branches and the base of Diptera are shown.

Branch (from node..to node)	Distance [aa subs/site]	Time^a ^[My]	Rate [10^-3 ^aa subs/site/My]	Relative rate^b^
*Tribolium *(1..4)	0.180 ± 0.003	280.0 ± 4.4	0.643 ± 0.015	---
*Anopheles *– base (3..4)	0.222 ± 0.003	280.0 ± 4.4	0.793 ± 0.016	1.233 ± 0.038
*Drosophila *– base (2..4)	0.245 ± 0.003	280.0 ± 4.4	0.875 ± 0.017	1.361 ± 0.041
*Anopheles *(3..5)	0.145 ± 0.002	241.0 ± 4.0	0.602 ± 0.013	0.936 ± 0.030
*Drosophila *(2..5)	0.168 ± 0.002	241.0 ± 4.0	0.697 ± 0.014	1.084 ± 0.033
Base of Diptera (4..5)	0.077 ± 0.002	39.0 ± 5.9	1.974 ± 0.303	3.071 ± 0.477

^a ^Age of geological stage as defined in "A Geologic Time Scale" [39] (see text for details).

^b ^Relative rate = Rate/(*Tribolium *Rate)

In order to assign absolute rates of evolution, we combined the sequence distances in Figure 1 with absolute dates obtained from palaeontological estimates. Based on the oldest coleopteran fossil [12] and the timing of the primary radiation of holometabolous insect orders [13], the divergence of dipterans (*Drosophila *and *Anopheles*) from coleopterans (*Tribolium*) has been estimated to the early Permian (the Artinskian; about 284.4-275.6 Mya). The divergence between the Brachycera (*Drosophila*) and the Nematocera (*Anopheles*) lineages has been estimated to the Middle Permian (the Anisian; about 245.0-237.0 Mya) [14]. Combining these dates with the maximum-likelihood estimates of branch lengths, we obtained the absolute evolutionary rates listed in Table 1. This shows that the 39 My time interval starting with the separation of coleopterans and dipterans and culminating in the radiation of Diptera was characterised by an episodic increase of evolutionary rate, averaging 3.07 times the mean rate found for the *Tribolium *lineage (95% CI: 2.39–4.34).

### Deviations from clock-like evolution

The accuracy of absolute rate estimates depends on the completeness of the known fossil record. Thus, the uncertainty assigned to the dates in Table 1, which reflects the uncertainty in the dating of the strata where fossils were found, is an underestimate of the true uncertainty. To confirm that the dipteran rate acceleration is not an artefact of erroneous palaeontological estimates, we performed additional analyses that directly compared relative evolutionary rates. To this end, we implemented several models involving global or local molecular clocks [15]. Because these models are nested, they can be compared via likelihood-ratio tests [16]. Considering the branch length differences in Table 1, it is not surprising that the model employed above (without a clock assumption) fits the data much better than a model with a global clock, which enforces a constant rate of evolution across all lineages (*P *< 10^-66^, Table 2). Is the clock assumption violated only at specific (local) branches? Table 1 assigns the strongest rate acceleration to the base of the Diptera. However, an evolutionary model involving a local clock for this branch (with all other branches evolving at the same rate) still fits the data significantly worse than the model without a clock (*P *< 10^-12^, Table 2); the same is indeed true for all models involving a single local clock (data not shown). Thus, a burst of accelerated evolution at the base of the dipterans is not sufficient to explain the rate increase found for dipterans as a whole.

**Table 2:** Maximum likelihood estimates of different molecular clock models for concatenated sequences. The models are compared to the model without a clock via likelihood ratio tests.

**Clock model**	**Parameters**	**ln(L)**	**2[ln(L_nc_)-ln(L)]^a^**	**df^b^**	***P***
No clock	8	-459144	-	-	-
Global	5	-459296	302.89	3	2 × 10^-65^
Base of Diptera	6	-459229	169.93	2	1 × 10^-37^
*Drosophila *and *Anopheles*	7	-459203	117.57	1	2 × 10^-27^
*Tribolium *and *Drosophila*	7	-459146	2.69	1	0.1
*Tribolium *and *Anopheles*	7	-459150	10.60	1	0.001
*Tribolium *and Diptera	7	-459170	52.21	1	5 × 10^-13^
*Tribolium *and base of Diptera	7	-459170	52.21	1	5 × 10^-13^
*Tribolium *and tips of Diptera	7	-459170	52.21	1	5 × 10^-13^

^a ^L_nc _is the maximum-likelihood estimate of the model without a molecular clock.

^b ^df = degrees of freedom

Further comparison of increasingly complex models of evolution show that only models involving separate local clocks for the *Tribolium *and *Drosophila *lineages can fit the data as good as the model without a clock assumption (*P *= 0.1, Table 2). All other combinations of local clocks between the *Tribolium*, *Drosophila *and *Anopheles *lineages were found to be inferior to the model without the clock (*P *< 0.01, Table 2 and data not shown). Thus, although the accelerated evolution of dipterans was most pronounced during a burst of changes that occurred at the base of the order (Table 1), a sustained increase is also detected in the branches separating *Drosophila *from *Anopheles*. Remarkably, the need for a coleopteran local clock also demonstrates that the *Tribolium *lineage has generally evolved much slower than most of the other taxa considered here.

It should be noted that Table 2 contains multiple statistical comparisons, thereby decreasing the overall specificity of the statistical test. A conservative strategy to control for this is to divide the *P*-value cutoff for significance by the number of comparisons (Bonferroni-correction), i.e., to use *P*_0 _= 0.05/8 = 0.0063, or – if accounting for all comparisons in data not shown – P_0 _= 0.05/41 = 0.0012. This correction does not affect our conclusions.

### The dipteran acceleration affects the majority of individual genes

**Figure 2:**
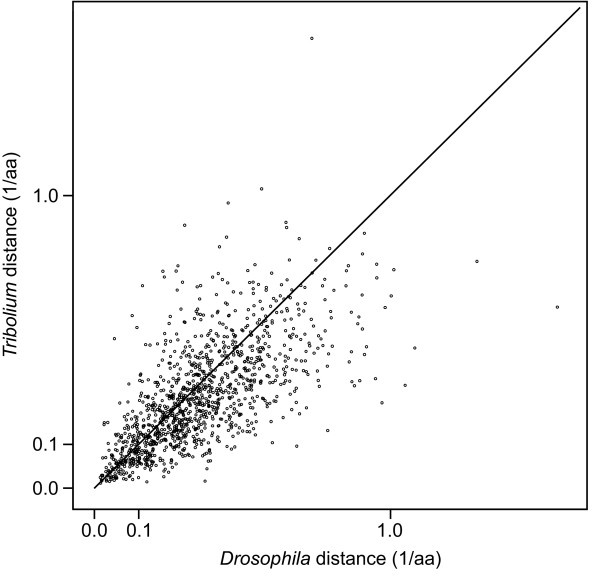
**Amino acid distances of 1199 individual *Tribolium *and *Drosophila *genes to their last common ancestor**. Comparison to the identity line expected under equal rates demonstrates that the acceleration is a genome-wide effect.

By analysing concatenated amino acid sequences, we have demonstrated accelerated evolution in dipterans, compared to a slower rate in the *Tribolium *lineage. Is this rate change confined to certain genes, or does it extend across the genome as a whole? To analyse this issue, we calculated branch lengths for 1199 orthologous groups comprising sequences from *Tribolium *and *Drosophila*, using human as an outgroup. In Figure 2, we compare the amino acid distances of *Tribolium *and *Drosophila *proteins to their last common ancestor. There is a very strong relationship between these *a priori *unrelated distances (Spearman's rank correlation coefficient *r*_s _= 0.718, *P *< 10^-3^).

When calculated from single gene distances, the mean evolutionary rate of the *Drosophila *lineage is significantly larger than the one of the *Tribolium *lineage (0.284 > 0.245; two-tailed Mann-Whitney *U-*test, *P *< 10^-3^). The corresponding constant of proportionality is 1.31, in close agreement with the relative rate increase of 1.36 obtained from the concatenated sequences (Table 1). Thus, although evolutionary rates among genes of a genome can vary by several orders of magnitude, these rates are nevertheless correlated between species even after more than 280 My of independent evolution.

## Discussion

Using 439 nuclear transcripts, we find that the dipteran lineage (represented by *Drosophila *and *Anopheles*) has experienced an episodic increase in evolutionary rate when compared to the coleopteran lineage (represented by *Tribolium*). This rate subsequently dropped in the diversifying dipteran lineage, but remained above average in *Drosophila*. This is consistent with previous findings from studies of ribosomal RNAs [9,17]. The rate increases found in our genome-scale analysis of protein sequences are lower than those reported for rRNAs, with an average increase of only 1.3-fold during dipteran evolution versus an at least threefold average increase for rRNAs [9]. However, our analysis is necessarily biased towards parts of genes that are sufficiently conserved to be detected in the outgroup (human) and that could be unequivocally aligned. Still, Figure 2 demonstrates that the averaged rate increase is representative for a large range of genes.

The Neutral Theory of Evolution [1,18,19] predicts that the accumulation of molecular changes is only driven by the mutation rate and the degree of purifying selection. Accordingly, genome-wide variation in the rate of protein evolution between species might be caused by physiological and ecological factors that affect the mutation rate (e.g. metabolic rate [20,21], temperature [20]), or by differences in the efficiency of selection (notably variation in effective population size [22-24] or the level of outbreeding [25]). In the case of the rRNA comparisons, we could indeed show that there has been a mutational bias towards incorporating more A/T nucleotides than G/C nucleotides at the base of the Diptera [9], which has probably caused the episodic acceleration of evolutionary rates. Such a mutational bias is likely to have affected the whole genome. However, the GC content of the *Tribolium *genes in this study (46.0%) is very close to that of the *Drosophila *orthologs (47.1%), suggesting that mutational biases are unlikely to be responsible for the continued acceleration in dipterans. This interpretation is supported by the fact that it is the *Tribolium *proteins which show a slight bias towards AT-rich amino acids when compared to the *Drosophila *orthologs (28.1% AT-rich amino acids for *Tribolium*, compared to 27.2% for *Drosophila*).

We observe a strong correlation between evolutionary rates in the *Tribolium *and the *Drosophila *lineages (Figure 2). This indicates that the selective forces acting on the majority of these genes have been similar between the two lineages, consistent with a neutral model. The correlation would be expected to become weaker or even disappear if positive selection would occur episodically among these genes. Our finding that most proteins diverge in a clock-like fashion, with clock speeds that differ among lineages only due to genome-wide effects, suggests that episodic changes are an exception, although such changes are known to occur in some cases [26,27]. However, we note that the class of fast evolving genes could not be analysed here, as they diverge too fast to allow identification as orthologs in distant comparisons [28]. In a dedicated study of such genes between closely related *Drosophila *lineages, we found that there are indeed a significant number of genes that must have undergone episodic changes in substitution rates on a gene by gene basis [29]. Thus, we emphasize that the above conclusions relate only to relatively conserved (or non-orphan) genes, while the generalized evolutionary patterns of fast evolving (or orphan) genes will need further study.

## Conclusion

We reported here the analysis of evolutionary rate variation of a large number of orthologous genes between insect lineages. Variation in the rate of evolution has genome-wide effects, and is correlated between orthologous genes over very long evolutionary time scales.

Because *Drosophila melanogaster *is among the best studied animal model organisms, the peculiar evolutionary pattern confirmed here has practical implications. In BLAST searches of human sequences against *Tribolium *and *Drosophila*, *Tribolium *sequences are found in 70% of cases to be more similar to human than *Drosophila *genes (*N *= 1221, *P *= 10^-50 ^from sign test). Thus, when attempting to link human genes to their *Drosophila *homologs, data from *Tribolium *will be helpful to provide a more conservative reference sequence. This approach has been used, e.g., to resolve the evolutionary relationship between the *Drosophila zen *gene and human HOX3 genes [30]. The slowly evolving beetle *Tribolium castaneum *is thus likely to play a significant role for comparisons between phyla.

## Methods

### Sequences

*Drosophila melanogaster*, *Anopheles gambiae *and *Homo sapiens *peptides were obtained from Ensembl [31]. For *Tribolium castaneum*, EST data available through NCBI dbEST [32] were assembled into contigs using *phrap *and manually curated to ensure high quality of the data set. For the pea aphid *Acyrthosiphon pisum*, a publicly available EST-based unigene set was obtained from the INFOBIOGEN GnpSeq database [33]. *Tribolium *and *Acyrthosiphon pisum *contigs were then searched against all *Drosophila melanogaster *proteins using BLASTx. The reading frame from the best hit was assumed to be the correct reading frame. We then chose the longest run of peptides uninterrupted by a stop codon as the peptide corresponding to each EST contig.

### Identification of orthologs

We performed BLASTp searches of all proteome pairs among *Tribolium castaneum, Drosophila melanogaster*, *Anopheles gambiae*, *Acyrthosiphon pisum *and *Homo sapiens*. Orthologs were selected based on reciprocal best blast hits [34] using an e-value cut-off of 10^-10^. A group of sequences with exactly one member in each species was accepted as an orthologous family if each sequence had each of the other family sequences as the best BLASTp hit in the respective proteome. This requirement of all-against-all reciprocal best hits is very stringent, and thus gives good confidence in the inferred orthology.

### Alignments and distance estimation

Multiple sequence alignments were performed with MUSCLE [35] using default settings. Resulting alignments were purged from putatively misaligned positions as well as gap positions with Gblocks using default settings [36].

Branch lengths were calculated with the maximum likelihood model by Goldman and Yang [11] as implemented in the PAML package [37]. We used the empirical transition matrix compiled by Jones et al. [38]. The distribution of evolutionary rates across sites was approximated by a discrete Γ-distribution, with the shape parameter as an additional free parameter. When calculating rates for individual genes, we assumed a uniform rate across sites. Clusters containing genes with zero branch length were discarded from further analysis.

## List of abbreviations

df – degrees of freedom

My – million years

Mya – million years ago

rRNA – ribosomal RNA

## Authors' contributions

JS and MJL designed the study, carried out sequence comparisons, performed the statistical analysis, and drafted the manuscript. DT conceived the study, and participated in its design and coordination.
